# Spexin level in acute myocardial infarction in the emergency department

**DOI:** 10.5937/jomb0-39485

**Published:** 2023-08-25

**Authors:** Yahya Çiftçi, Mehtap Gurger, Evrim Gul, Mustafa Yilmaz, Selda Telo, Metin Atescelik, Goktekin Mehmet Cagri, Kobat Mehmet Ali

**Affiliations:** 1 Gaziantep Sehitkamil State Hospital, Department of Emergency Medicine, Gaziantep, Turkey; 2 Firat University, Faculty of Medicine, Department of Emergency Medicine, Elazig, Turkey; 3 Firat University, Faculty of Medicine, Biochemistry and Clinical Biochemistry, Elazig, Turkey; 4 Firat University, Faculty of Medicine, Department of Cardiology, Elazig, Turkey

**Keywords:** spexin, acute myocardial infarction, diagnosis, speksin, akutni infarkt miokarda, dijagnoza

## Abstract

**Background:**

We aimed to determine the serum spexin level in patients with acute myocardial infarction (AMI) admitted to the emergency department.

**Methods:**

A total of 100 patients with AMI (50 with ST-segment elevation myocardial infarction (STEMI) and 50 with non-ST-segment elevation myocardial infarction (NSTEMI)) and 50 control group patients with non-cardiac chest pain were included in the study. A detailed anamnesis was taken, a physical examination was performed, and 12-lead electrocardiograms and venous blood samples were taken at the time of admission. Spexin levels were measured via enzyme-linked immunosorbent assay.

**Results:**

Serum spexin levels were significantly lower in the AMI group than in the non-cardiac chest pain group (p<0.001). There was no significant difference in serum spexin levels between STEMI and NSTEMI patients (p=0.83). In receiver operating curve analysis, we detected 58% sensitivity, 76% specificity, 82.9% positive predictive value, and 47.5% negative predictive value with an optimal cutoff value of 532 pg/mL for the diagnosis of AMI.

**Conclusions:**

In this study, serum spexin levels were significantly lower in AMI patients compared to patients with non-cardiac chest pain. The decrease in spexin levels suggests that it has the potential to be used as a diagnostic marker in AMI patients.

## Abstract (sr)

**Uvod:**

Naš cilj je bio da odredimo nivo speksina u serumu kod pacijenata sa akutnim infarktom miokarda (AMI) koji su primljeni na odeljenje urgentne medicine.

**Metode:**

U studiju je uključeno ukupno 100 pacijenata sa AMI (50 sa infarktom miokarda sa elevacijom ST segmenta (STEMI) i 50 pacijenata sa infarktom miokarda sa elevacijom non-ST segmenta (NSTEMI)) i 50 pacijenata kontrolne grupe sa bolom u grudima čiji uzrok nisu bili problemi sa srcem. Uzeta je detaljna anamneza, urađen je klinički pregled, a prilikom prijema su uzeti 12-kanalni elektro - kardio g rami i uzorci venske krvi. Nivoi speksina su mereni putem enzimskog imunosorbentnog testa.

**Rezultati:**

Nivoi speksina u serumu su bili značajno niži u grupi sa AMI nego u grupi koja nije imala srčani bol u grudima (p<0,001). Nije bilo značajne razlike u nivoima speksina u serumu između pacijenata sa STEMI i NSTEMI (p=0,83). U analizi ROC krive, smo detektovali 58% osetljivosti, 76% specifičnosti, 82,9% pozitivne prediktivne vrednosti i 47,5% negativne prediktivne vrednosti sa optimalnom graničnom vrednošcu od 532 pg/mL za dijagnozu AMI.

**Zaključak:**

U ovoj studiji, nivoi speksina u serumu su bili značajno niži kod pacijenata sa AMI u poređenju sa pacijentima sa bolom u grudima čiji uzrok nisu bili problemi sa srcem. Smanjenje nivoa speksina ukazuje da on ima potencijal da se koristi kao dijagnostički marker kod pacijenata sa AMI.

## Introduction

One of the most common reasons for admitting patients to the emergency department is chest pain [1]. Approximately 10–20% of patients admitted with chest pain are diagnosed with acute coronary syndrome (ACS) [2], while more than half have non-cardiac causes [1] [3]. Because of the significant risk of mortality and morbidity in ACS patients, early detection and prompt initiation of treatment are critical, but it is essential to rule out causes of chest pain that do not require immediate medical attention [4]. When evaluating patients who apply to the emergency department with chest pain complaints, life-threatening causes are primarily determined, and the focus is on stabilizing the patient. While evaluating the patients, a detailed anamnesis is taken, a physical examination is performed, and electrocardiograms (ECG) are taken within the first 10 minutes and interpreted in line with the recommendations of current guidelines. Along with these clinical data, some biochemical markers are used to detect or exclude myocardial damage. Cardiac troponins are the most sensitive tests used to detect acute myocardial injury. Although troponins are organ-specific, they are not disease-specific and are elevated in myocardial damage due to non-coronary causes [3].

Spexin is a recently identified peptide [5]. Spexin, consisting of 14 amino acids, is widely found in peripheral tissue and the central nervous system [6]. Spexin, believed to have a regulatory role in energy metabolism, is associated with cardiovascular risk factors such as obesity, hypertension, and diabetes [7]. In this study, we aimed to investigate the serum spexin level in patients diagnosed with acute myocardial infarction (AMI) in the emergency department.

## Materials and methods

A total of 100 patients diagnosed with AMI due to the evaluation and 50 patients in the control group with non-cardiac chest pain were included in this study. A detailed anamnesis was taken from all participants, a physical examination was performed, and a 12-lead ECG was taken within 10 minutes of admission. AMI was diagnosed with typical symptoms consistent with myocardial ischemia, newly developed ischemic ST-T changes in at least two adjacent ECG leads, and elevation of cardiac troponin I level with at least one value above the 99^th^ percentile of the upper reference limit. According to ECG, patients diagnosed with AMI, ST-segment elevation myocardial infarction (STEMI) (>20 minutes), and non-ST-segment elevation myocardial infarction (NSTEMI) (n=50) (transient ST-segment elevation, ST-segment depression, T wave inversion) were divided into two groups. The control group, who presented to the emergency department with chest pain, consisted of patients without STEMI, NSTEMI, unstable or stable angina pectoris but with non-cardiac chest pain (musculoskeletal pain, panic attack, pneumonia, gastroesophageal disorders) as a result of clinical, ECG and troponin evaluation. Patients with concomitant cerebrovascular disease, renal and hepatic failure, trauma, venous thromboembolism, and peripheral arterial occlusion were excluded from the study. For this study, written informed consent forms were obtained from all participants, and the Helsinki declaration was made following the ethical guidelines. Ethics committee approval was received from Fırat University Ethics Committee (No: 2020/02-26).

### Laboratory analysis

Venous blood samples for troponin and spexin levels were obtained during admission to the emergency department. Troponin I level was studied using the AQT90 FLEX (Radiometer Medical ApS, Denmark) analyzer using fluorometric detection. Blood samples for spexin levels were centrifuged (3000 rpm, 10 min), and the separated serum was stored at -80°C until the study day. Spexin level was determined using the Enzyme-linked immunosorbent assay (ELISA) method with kits (Bioassay Technology Laboratory-BT Lab, China; Human Spexin ELISA kit, Cat. No: E3507Hu) following kit procedures. The measured values were recorded in pg/mL. Sensitivity for Spexin was found to be 4.95 pg/mL, intra-assay CV<8%, and inter-assay <10%.

### Statistical analysis

Data were analyzed using the Statistical Package for the Social Sciences (SPSS 22, Chicago, IL, USA)statistical program. The normality of data distributions was examined using the Kolmogorov–Smirnov test. Variables were expressed mean±standard deviation, Median (IQR: interquartile range), and percentages. The Kruskal-Wallis test was used to compare groups. The Mann-Whitney U test was used to determine the relationship between the groups. The Chi-Square test (cross-tab) was used to compare categorical data. The Spearman correlation test was used to evaluate the correlation of Spexin level with ordinal variable scales. The receiver operating characteristic (ROC) curve analysis was performed to assess the ability of spexin level to predict AMI. P<0.05 was considered significant.

## Results

**Figure 1:**
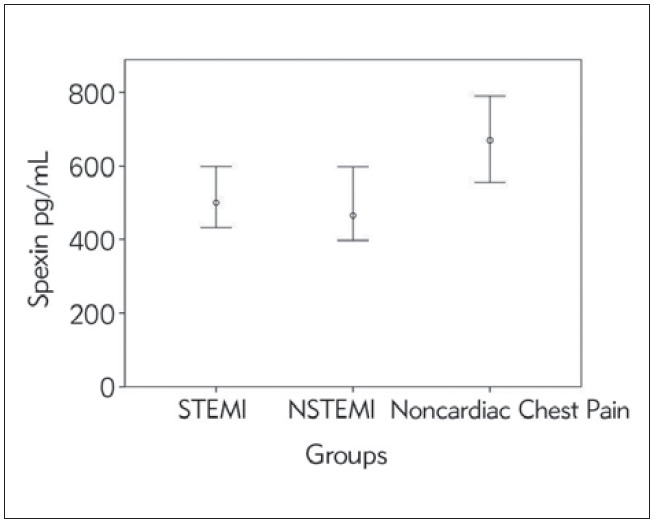
Spexin levels in the groups with STEMI, NSTEMI, and non-cardiac chest pain.

A total of 100 AMI patients, 50 with STEMI and 50 with NSTEMI, were included in the study, while fifty patients with non-cardiac chest pain were in the control group. The serum spexin levels of the groups are given in Figure 1, and the demographic and laboratory findings of the groups are given in Table 1. Serum spexin was significantly lower in the STEMI (p<0.001) and NSTEMI (p<0.001) groups compared to the group with non-cardiac chest pain. There was no significant difference in serum spexin levels between STEMI and NSTEMI patients (p=0.83).

**Table 1:** Demographic, clinical, and laboratory data of groups. BMI: Body mass index, IQR: interquartile range

	STEMI<br>n=50	NSTEMI<br>n=50	Noncardiac Chest Pain<br>n=50
Age (Years)	62.1±11.2	64.5±10.6	43.3±15.2
Gender (Female/Male)	10/40	11/39	13/37
BMI (kg/m^2^)	27.6±3.2	28.3±4.3	25.7±2.8
Troponin median (IQR)<br>Range: 0.010–0.023, μg/L	0.05 (0.01–0.14)	0.25 (0.14–0.83)	0
Spexin median (IQR), pg/mL	500 (345–679)	465 (337.3–667.5)	670 (529.5–914.3)
Underlying Disease n(%)<br>-Diabetes Mellitus<br>-Hypertension<br>-Dyslipidemia	<br>23 (46)<br>36 (72)<br>22 (44)	<br>24(48)<br>43 (86)<br>40(80)	<br>3(6)<br>10 (20)<br>9 (18)

**Figure 2:**
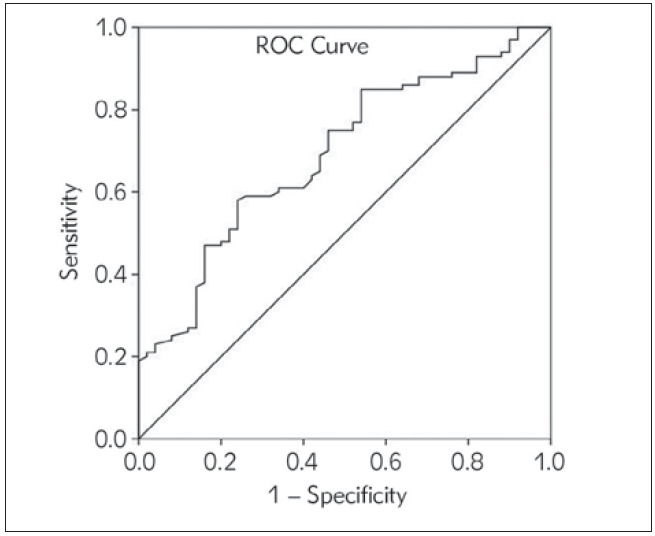
Receiver operating characteristic curve analysis showing the performance of spexin level in the diagnosis of acute myocardial infarction.

In the ROC analysis, the optimal cutoff value of 532 pg/mL of spexin was associated with 58% sensitivity, 76% specificity, 82.9% positive predictive value (PPV), and 47.5% negative predictive value (NPV) for the diagnosis of AMI (Area Under the Curve (AUC): 0.692, 95% confidence interval (CI): 0.611–0.764, p<0.0001)) (Figure 2).

In our study, when the spexin levels of the patients diagnosed with AMI were compared according to the underlying diseases, the spexin level was lower in diabetic patients than in nondiabetic patients with AMI. However, no significant difference was observed between patients with hypertension and hyperlipidemia (Table 2). The mean spexin level (540 pg/mL) in nondiabetic AMI patients was significantly lower than that of non-cardiac chest pain (670 pg/mL) (p=0.012).

**Table 2:** Serum spexin level in the AMI group according to the underlying disease. IQR: interquartile range

Underlying<br>Disease	Exist	Absent	p
Diabetes Mellitus<br>Median (IQR)	n=47<br>421 (255–599)	n=53<br>540 (430–701)	0.012
Hypertension<br>Median (IQR)	n=79<br>478 (345–655)	n=21<br>532 (332–750)	0.630
Dyslipidemia<br>Median (IQR)	n=62<br>498 (368–657)	n=38<br>472 (289–714)	0.966

The median admission time in STEMI patients was 2 hours (IQR: 1–3 hours), whereas it was 6 hours (IQR: 3–12 hours) in the NSTEMI group. There was no significant correlation between the onset of chest pain and spexin level (r=-0.159, p=0.14). There was a negative correlation between spexin level with patients’ age (r=-0.24, p=0.003) and body mass index (BMI) (r=-0.255, p=0.002).

## Discussion

In this study, we found that serum spexin level was significantly lower in STEMI and NSTEMI groups than in non-cardiac chest pain. There was no significant difference between the spexin levels of the AMI groups with and without ST-segment elevation.

Spexin is a peptide that is broadly expressed in many different tissues of the body. It has been asserted to regulate energy metabolism and regulate metabolic disorders [6]. Spexin has also been reported to have a critical role in lipid and glucose metabolism, stimulate lipolysis on adipocytes, have an inhibitory effect on glucose uptake and lipogenesis, and be negatively correlated with blood glucose level, hemoglobin A1C, triglycerides, and LDL-C levels in type 2 diabetes patients [8] [9]. On the other hand, the level of spexin has been shown to decrease in diseases such as hypertension [10] [11], obesity [10] [12] [13], metabolic syndrome [14], and diabetes [8] [9] [12] [15] that may cause cardiovascular diseases. In addition, studies have reported a negative correlation between age [12] and BMI [6] [10], which are prominent cardiovascular risk factors, with the serum spexin level. In our study, there was a negative correlation between the spexin levels with the age of the patients and BMI. In this study, the serum spexin level of patients with diabetic AMI was significantly lower than patients with nondiabetic AMI. However, no significant difference was found between patients with hypertension and hyperlipidemia.

Apart from its metabolic role, spexin has also been reported to play a role in inflammation [16] [17] [18]. Gambaro et al. [16] showed that spexin caused a decrease in the expression of proinflammatory markers such as IL1 , IL6, and TNF but an improvement in anti-inflammatory markers such as IL10 and CD206 in obese rats. A positive correlation between spexin and serum levels of IL-1 and IL-10 has been reported [17]. Stating that there is a negative correlation between spexin and leptin in adolescents with obesity and that hs-CRP concentration is higher at low spexin and increased leptin levels, Kumar et al. [18] emphasized that spexin may have a potential role in the regulation of cardiovascular risk factors in children. It is known that inflammation is important in the pathogenesis of cardiovascular diseases [19]. Therefore, spexin may play a role as a biomarker in cardiovascular diseases considering its effects on metabolic events and inflammation.

The exact role of spexin in cardiac energy metabolism is not known. Liu et al. [7] demonstratedthat it is expressed in heart tissue in humans and mice, and the level of spexin decreases in cardiomyocytes of mice exposed to hypoxia. In mice treated with spexin before hypoxia, spexin has been shown to promote fatty acid metabolism during hypoxia, protect mice heart tissues from severe mitochondrial damage, prevent hypoxia-induced ATP decline, limit the production of reactive oxygen species, and thus increase ATP levels in cardiomyocytes. In this study, they found that spexin preserves the energy and mitochondrial homeostasis of cardiomyocytes, thus suggesting that spexin may play a potential role in the treatment of cardiovascular diseases. As far as we know, there is no study investigating the level of spexin in AMI patients. Significant changes occur in cardiac energy metabolism during AMI. In addition, sudden ischemia of the heart suppresses the metabolism of carbohydrates, fatty acids, amino acids, and ketones, leading to changes in cardiac metabolism [20]. Liu et al. [7] reported that decreased spexin during hypoxia indicates intracellular depletion. Our study showed that serum spexin levels decreased in AMI patients. Accordingly, the decrease in spexin level suggests that it may protect cardiac energy metabolism.

In conclusion, to the best of our knowledge, this study presented the first report showing decreased serum spexin levels in AMI patients. Our current findings are promising for detecting AMI of serum spexin levels. More extensive studies are needed to confirm our findings and explain the underlying mechanisms.

Our study has some limitations. First, in our study, the level of spexin was measured only at thetime of admission, and serial measurements of spexin could not be performed. In addition, we did not examine the prognostic value of spexin for the level of unstable and stable angina pectoris and major adverse cardiac events.

## Dodatak

### Research Funding

This study was supported by Fırat University Scientific Research Projects Coordination Unit (FUBAP: TF.20.41).

### Conflict of interest statement

All the authors declare that they have no conflict of interest in this work.
